# Inadequate blood pressure control, increasing challenge in dementia prevention among older adults of Nepal: Preliminary findings

**DOI:** 10.1002/alz70860_099620

**Published:** 2025-12-23

**Authors:** Prabha Shrestha, Victor Valcour, Biraj Man Karmacharya, Yunika Acharya, Indu Poudel

**Affiliations:** ^1^ Kathmandu University School of Medical Sciences, Dhulikhel, Bagmati, Nepal; ^2^ Global Brain Health Institute, University of California, San Francisco, CA, USA; ^3^ Memory and Aging Center, University of California San Francisco, San Francisco, CA, USA; ^4^ Global Brain Health Institute, University of California, San Francisco, San Francisco, CA, USA; ^5^ Research and Development Division, Dhulikhel Hospital‐Kathmandu University Hospital, Nepal, Dhulikhel, Bagmati, Nepal; ^6^ Research and Development Division, Dhulikhel Hospital‐Kathmandu University Hospital, Nepal, Dhulkhel, Bagmati, Nepal

## Abstract

**Background:**

Hypertension is common among older adults in Nepal, with poor control increasing the risk of stroke, heart disease, and dementia. Medication adherence is at 50%, and uncontrolled hypertension affects 31% to 67% of individuals. This study aimed to assess the prevalence of uncontrolled hypertension and its association with medication adherence, BMI, comorbidities, and living arrangements in older adults in Dhulikhel Municipality, Nepal.

**Method:**

We conducted a cross‐sectional study among hypertensive older adults aged 60 years and above. Samples were randomly selected from the sampling frame of self‐reported hypertension within the “Healthy City Initiative” data set. The Face to face interviews were conducted, and medication adherence was measured using the Hill Bone Medication Adherence Scale, scores ranged from 9 to 36, with higher scores indicating lower adherence. Systolic BP>140 mmHg and diastolic BP >90 mmHg representing uncontrolled hypertension. We included data from the first 101 participants enrolled in this interim analysis. We excluded participants with cognitive impairment, those who were unreachable, uninterested, or deceased. Logistic regression assessed the associations between hypertension control and the stated factors, adjusting for socio‐demographic variables using Stata v13.

**Result:**

Over half of the participants had uncontrolled hypertension (54%). The mean medication adherence score was 10.6 (2.04). Most (51%) of participants were overweight or obese. After adjusting for socio‐demographic variables including age, sex, educational status, ethnicity, and religion, no significant associations were found between hypertension control and medication adherence (*p* = 0.6), BMI (*p* = 0.8), presence of comorbidity (*p* = 0.7), or living arrangement (*p* = 0.3).

**Conclusion:**

The frequency of uncontrolled hypertension is high among hypertensive older adults in Dhulikhel Municipality, highlighting gaps in effective management. Despite moderate medication adherence and high obesity rates, these factors were not significantly linked to hypertension control. Further research is needed to identify other factors for improving blood pressure control and reducing cardiovascular and dementia risks in Nepal.

